# Room temperature synthesis of a luminescent crystalline Cu–BTC coordination polymer and metal–organic framework†

**DOI:** 10.1039/d1ma00866h

**Published:** 2021-11-22

**Authors:** Shiraz Ahmed Siddiqui, Alexander Prado-Roller, Hidetsugu Shiozawa

**Affiliations:** Faculty of Physics, University of Vienna Boltzmanngasse 5 1090 Vienna Austria hidetsugu.shiozawa@univie.ac.at hide.shiozawa@jh-inst.cas.cz; Department of Inorganic Chemistry, University of Vienna Währinger Straβe 42 1090, Vienna Austria; J. Heyrovsky Institute of Physical Chemistry, Czech Academy of Sciences Dolejskova 3 182 23 Prague 8 Czech Republic

## Abstract

Synthesis of crystalline materials is elemental in the field of coordination chemistry towards optical applications. In the present work, coordination between copper and benzene-1,3,5-tricarboxylic acid (BTC) is controlled by adjusting the pH scale of the reaction mixture at room temperature to synthesize two crystalline structures: metal–organic framework HKUST-1 and coordination polymer Cu(BTC)·3H_2_O. The post-synthesis transformation of HKUST-1 into Cu(BTC)·3H_2_O is further demonstrated. Single crystals of both structures are studied by multi-laser Raman and luminescence spectroscopy. It is found that both crystals exhibit photoluminescence in the range of 700–900 cm^−1^ within the optical gap of the bulk materials, which can be associated with crystallographic defects. This work gives impetus for the synthesis of large metal–organic crystals based on which optical properties can be studied in depth.

## Abstract 2

Synthesis of crystalline materials is elemental in the field of coordination chemistry towards optical applications.

## Graphical abstract

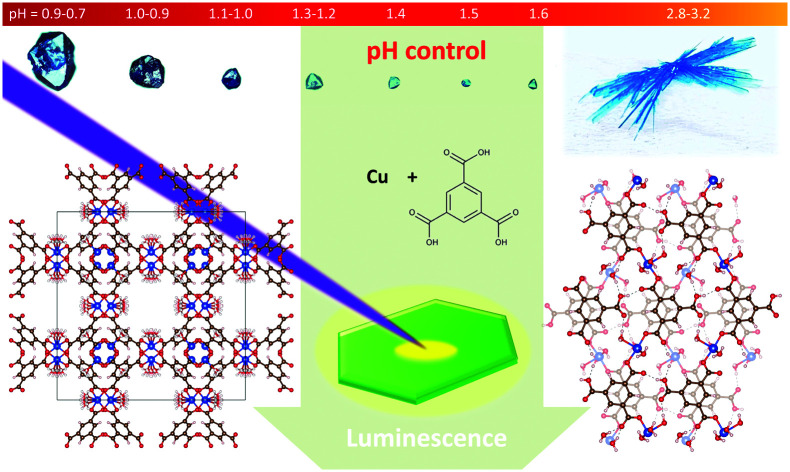

## 1 Introduction

Organic coordination compounds consist of organic ligands (Lewis base) bound to a central metal ion (Lewis acid).^1–4^ The unique and diverse properties of coordination compounds have been proved to be beneficial in the field of electronics, sensors, magnetism and medical research. Coordination polymers are solid-state structures consisting of repeating coordination units to form 1D extended chains, 2D sheets or 3D frameworks.^5–8^ Coordination polymers having a porous structure are known as metal–organic frameworks (MOFs).^9–16^ Synthesizing large and quality crystals of MOFs benefits in a wide range of applications such as separation, heterogeneous catalysis,^17–19^ gas-sorption,^20,21^ chromatography,^22,23^ optoelectronics, magnetism and sensing applications.^24,25^

Controlling the process of crystallization by pH adjustment is a challenging, yet fascinating process in understanding the chemistry of materials.^26–28^ The pH modulating technique was previously utilized to synthesize MOFs.^29–34^

**Fig. 1:**
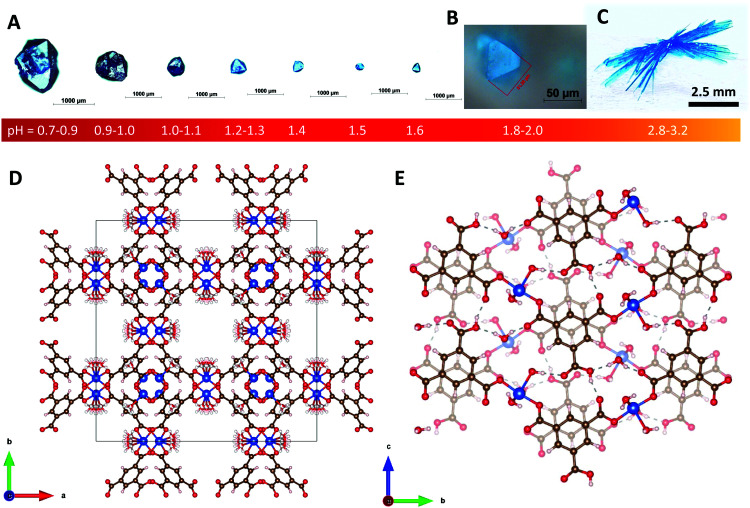
(A) Seven HKUST-1 crystals synthesized with HNO_3_ in the pH range 0.7–1.6. (B) HKUST-1 crystal synthesized without any pH adjustment. (C) Cu(BTC)·3H_2_O crystals synthesized in the pH range 2.8–3.2. (D) Crystallographic representation of HKUST-1. (E) Crystallographic representation of the Cu(BTC)·3H_2_O coordination polymer viewed along the [100] crystallographic axis.

In the present work, the reaction between copper and benzene-1,3,5-tricarboxylic (BTC) acid is controlled by adjusting the pH of the reaction mixture to obtain crystals of two different structures: Cu_3_(BTC)_2_(H_2_O)_3_ (also known as HKUST-1^35^ or MOF-199) and Cu(BTC)·3H_2_O (see Fig. 1).

HKUST-1 is typically synthesized using a hydrothermal/solvothermal method. The high-temperature synthesis yields larger crystals, but the problem is that a significant amount of Cu_2_O is obtained as a by-product. This by-product remains in the pores of the framework and reduces the specific surface area thereby causing hinderance in gas absorption.^36^ Thus, room-temperature synthesis is beneficial. Our pH-controlled synthesis procedure will help the community to synthesize quality and large crystals of HKUST-1 at room temperature.

In turn, copper(ii) based coordination polymers are some of the well studied compounds in the field of natural science and medical research. Owing to the antibacterial activity, low toxicity and potential as biomaterials, these compounds are gaining interest among researchers.^37^ In the present work, large crystals of Cu(BTC)·3H_2_O are synthesized at room temperature under weak acid conditions *i.e.* pH greater than 2.1. Single crystal X-ray diffraction (SXRD) reveals that the structure is composed of 1D zig-zag chains interconnected by hydrogen bonding which makes the structure stable and generates a layered coordination network.^38^

The knowledge acquired through the pH-controlled synthesis helps us transform the porous HKUST-1 framework into Cu(BTC)·3H_2_O. This process is particularly useful as MOFs sustain the porosity capable of absorbing harmful gases which can be a threat to the environment when the MOFs decompose. Our technique to use water is a fast, efficient and economical method of transforming the framework into non-porous coordination solids for safe disposal.

Finally, luminescent properties of both the HKUST-1 framework and Cu(BTC)·3H_2_O are examined on their single crystals with multiple visible lasers. It is found that both crystals luminesce in the range 700–900 cm^−1^ with a red excitation laser, that can be attributed to crystallographic defects.

## 2 Results and discussions

**Fig. 2:**
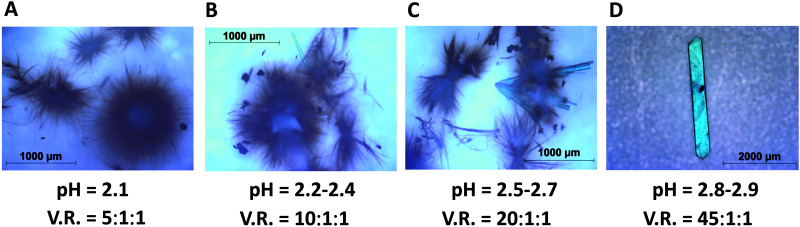
Crystals and whiskers of Cu(BTC)·3H_2_O synthesized at different pH scales. V.R. stands for the volume ratio of three solvents (deionised water, DMF, and ethanol).

All synthesis demonstrated in this work is done at room temperature. It is found that crystals of HKUST-1 can be synthesized by adjusting the pH of the reaction mixture in the pH range 0.7–1.6 as shown in Fig. 1A, whereas crystals of Cu(BTC)·3H_2_O can be synthesized in the pH range 2.2–3.2 as shown in Fig. 1C and 2B–D.

### 2.1 Synthesis of HKUST-1

First, we present room temperature synthesis of HKUST-1 (crystal size 50–55 micrometers) without additional pH adjustment. Copper nitrate trihydrate CuNO_3_·3H_2_O (1 mmol) and BTC (1 mmol) is dissolved in a mixture of three solvents (deionised water 4 ml, DMF 4 ml and ethanol 4 ml) *i.e.* in a volume ratio (V.R.) of 1 : 1 : 1 (pH = 1.8–2.0). After 16–20 hours, crystals of HKUST-1 are formed at the bottom of the vial (pH = 2.1). A clear depiction of the morphology of the as-synthesized HKUST-1 crystal is shown in Fig. 1B. The crystal size is approximately 50 μm across. The homogeneity range is shown in Fig. S1 in the ESI.† An SXRD analysis confirms the well-known structure of HKUST-1 MOF as depicted in Fig. 1D. See the ESI† for more details.

Much larger crystals can be obtained in more acidic conditions prepared by adding 0.1 ml of nitric acid (HNO_3_) in the aforementioned 12 ml reaction mixture of V.R. = 1 : 1 : 1. The resulting pH scale is in the range 0.7–0.9. After 8–10 days, crystals of HKUST-1 can get as large as ∼1–1.2 mm, as shown in Fig. 1A. More examples of such crystals are shown in Fig. S2 in the ESI.† The size can be controlled by adjusting the pH of the reaction mixture in the range from 0.7 to 2.0, as shown in Fig. 1A. Similarly, large crystals of HKUST-1 in the size range ∼0.9–1.0 mm can also be obtained by using 37% hydrochloric acid (HCl) instead of HNO_3_ and copper(ii) chloride (CuCl_2_) instead of CuNO_3_·3H_2_O. Their crystal size and morphology are shown in Fig. S3 in the ESI.†

It has been reported previously that large crystals of MOFs can be synthesized *via* slow reactions.^22,39^ Specifically, large crystals of HKUST-1 could be synthesized in acidic conditions.^23^ Deprotonation of BTC is a prerequisite for HKUST-1 growth. In our pH dependent synthesis, HKUST-1 crystals grow larger when a strong acid, HCl or HNO_3_, is added in the reaction mixture. The increased amount of H^+^ in the mixture inhibits the deprotonation of the BTC ligand. A reduced amount of BTC^3−^ anion leads to slowing down the nucleation and further growth of fewer crystals may be possible.

### 2.2 Synthesis of Cu(BTC)·3H_2_O

When the pH is adjusted to exceed 2.1 by adding excess water in the reaction mixture to get a volume ratio of V.R. = 5 : 1 : 1, another type of crystal is formed. Fig. 1C shows this large crystal obtained 2–3 weeks after mixing the precursors. SXRD analysis reveals the structure of Cu(BTC)·3H_2_O as shown in Fig. 1E. At pH = 2.1, tiny whiskers appear in 3–4 hours as shown in Fig. 2A. This indicates the point of nucleation. When the pH is increased to 2.2–2.4 (V.R. = 10 : 1 : 1), both tiny whiskers and bar-shaped crystals grow in 12–16 hours as shown in Fig. 2B. A further increase in pH to 2.5–2.7 (V.R. = 20 : 1 : 1) accelerates the crystal growth, resulting in more bar-shaped crystals observed in 16–20 hours as shown in Fig. 2C. At pH = 2.8–2.9 (V.R. = 45 : 1 : 1), the crystal growth is optimized for the formation of only large bar-shaped crystals as shown in the micrograph taken after 18–22 hours in Fig. 2D.

**Fig. 3:**
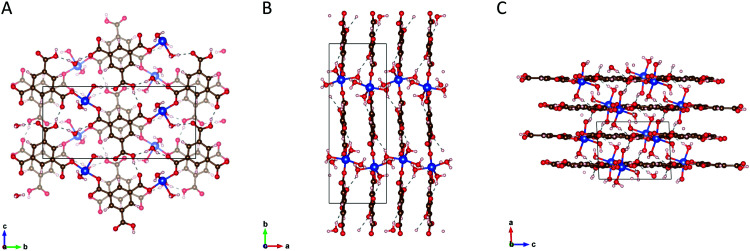
Panels (A, B and C) shows the structures of the Cu(BTC)·3H_2_O coordination polymer along the [100], [001] and [010] crystallographic planes, respectively.

HKUST-1 is not stable in this condition to be demonstrated as the transformation of HKUST-1 to Cu(BTC)·3H_2_O in Section 2.4. SXRD evidences that two of the three carboxyl groups of the BTC ligand are coordinated to the Cu^2+^ ion and form zig-zag chains that are linked *via* hydrogen bonding facilitated by the third uncoordinated/protonated carboxyl group and coordinated water molecules (see Fig. 3). The stability of the structure relies on hydrogen bonding of the copper-coordinated water molecules and the pi stacking of the BTC ligands.^38^

### 2.3 Structure of Cu(BTC)·3H_2_O

The crystallographic representations of the Cu(BTC)·3H_2_O packing structure along the [100], [001] and [010] crystallographic axes are shown in Fig. 3A–C, respectively. For further and detailed information regarding the structure of HKUST-1 and Cu(BTC)·3H_2_O, see Section S2 in the ESI.†

The crystal structure is monoclinic with space group *P*21/*n* and lattice parameters *a* = 6.7654, *b* = 18.8135, *c* = 8.5144; *α* = *γ* = 90°, *β* = 92.439°. The empirical formula of the structure is C_9_H_10_CuO_9_. This structure was previously reported.^37,38,40–42^Fig. 3A shows the 2D layer of Cu–BTC. Each copper is coordinated to two BTC and three water molecules in the distorted square pyramidal or pentacoordinate geometry.^38,42^ BTC consists of three carboxyl groups attached to the benzene ring. Two of the three carboxyl groups of the ligand are coordinated to the metal ion *via* one oxygen to form the zig-zag Cu–BTC polymeric chain. The other oxygen atoms contribute to the hydrogen bonding (dotted line) between the Cu–BTC chains. The Cu–BTC layers are held together by a combination of hydrogen bonding (dotted line) and π–π stacking of the BTC ligand (see Fig. 3B and C). The distance between the adjacent planes is approximately 3.4 Å.

### 2.4 Transformation of HKUST-1 to Cu(BTC)·3H_2_O

One of the major concerns in MOF research is that the porous framework can absorb harmful gases. These harmful gases trapped in the pores of the framework can cause threats to the environment. Our results strongly suggest that post-synthesis transformations between HKUST-1 and Cu(BTC)·3H_2_O can be possible by adjusting the pH of the reaction mixture. Indeed, when crystals of HKUST-1 are immersed in deionised water to get the pH range 2.8–3.2 suitable for obtaining Cu(BTC)·3H_2_O (see Fig. 2D), the HKUST-1 crystals transform into Cu(BTC)·3H_2_O in 18–22 hours. The reason behind this conversion is that the water molecule coordinates the metal site of the framework which results in increased coordination around the metal site. As a result, hydrolysis takes place and the organic ligand (BTC) is displaced away from the framework. Ultimately, the metal hydroxide remains along with the protonated linker.^42^

### 2.5 Raman spectroscopy

**Fig. 4:**
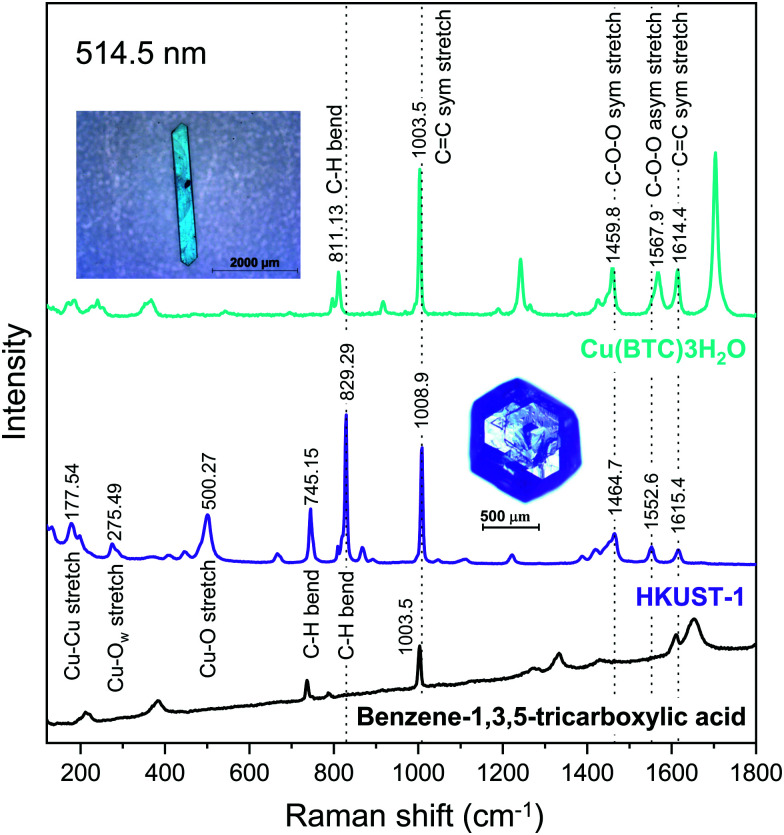
Raman spectra of HKUST-1 and Cu(BTC)·3H_2_O measured at *λ* = 514.5 nm. The insets show the measured single crystals of HKUST-1 and Cu(BTC)·3H_2_O.

Fig. 4 shows the Raman spectra of HKUST-1 and Cu(BTC)·3H_2_O measured with a green laser (wavelength *λ* = 514.5 nm) focused on the single crystals in the insets. For HKUST-1, the bands appearing in the low frequency region *i.e.* below 600 cm^−1^ is due to the copper ions in the framework. The doublet at 177.54 cm^−1^ and 197.63 cm^−1^ indicates that the MOF is hydrated and correspondingly Cu–Cu stretching modes appear.^43–45^ The band at 275.49 cm^−1^ is indicative of the Cu–O_w_ stretching vibration, where O_w_ represents the oxygen of the water molecule adsorbed on Cu^2+^ ions.

The bands in the range 700–1800 cm^−1^ are mostly due to vibrational modes of the BTC ligand. The C=C stretching modes of the benzene ring are found at 1008.9 and 1615.4 cm^−1^ for HKUST-1,^43,44,46–48^ and at 1003.5 and 1614.4 cm^−1^ for Cu(BTC)·3H_2_O. The peaks at 745.15 and 829.29 cm^−1^ for HKUST-1 can be attributed to the C–H bending modes.^43,44^ The corresponding peak for Cu(BTC)·3H_2_O is located at 811.13 cm^−1^. The peaks assigned to the C–O–O symmetric and C–O–O asymmetric stretching modes are centered at 1464.7 and 1552.6 cm^−1^, respectively, for HKUST-1,^48,49^ and at 1459.8 and 1567.9 cm^−1^ for Cu(BTC)·3H_2_O.

### 2.6 Photoluminescence spectroscopy

**Fig. 5:**
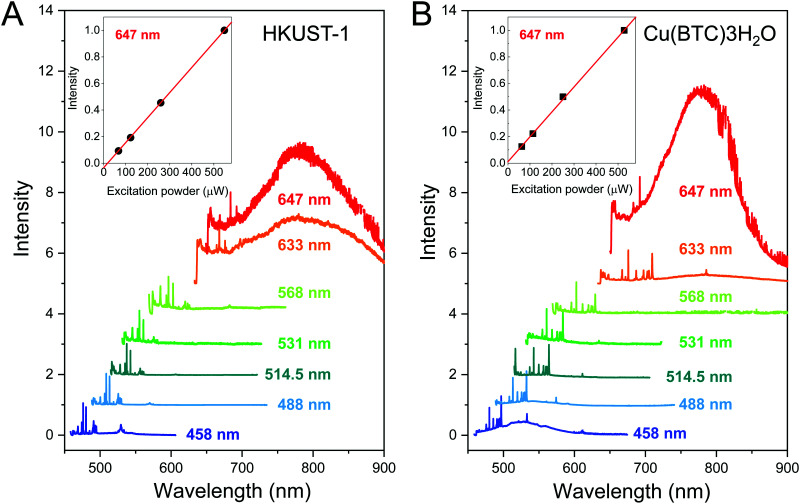
(A) Photoluminescence spectra (Raman spectra plotted as a function of wavelength) of a HKUST-1 crystal measured on the same spot using seven different lasers *λ* = 458, 488, 514.5, 531, 568, 633 and 647 nm with laser powers of 0.109, 3.78, 0.617, 0.0712, 1.10, 1.72 and 0.617 mW, respectively. All spectra were normalized by the highest Raman peak. (B) Photoluminescence spectra of a Cu(BTC)·3H_2_O crystal measured on the same spot using seven different lasers *λ* = 458, 488, 514.5, 531, 568, 633 and 647 nm with laser powders of 0.105, 1.37, 0.565, 0.077, 0.708, 1.20, and 0.815 mW, respectively. All spectra were normalized by the highest Raman peak. (insets) The photoluminescence intensity plotted against the laser power at *λ* = 647 nm.

**Fig. 6:**
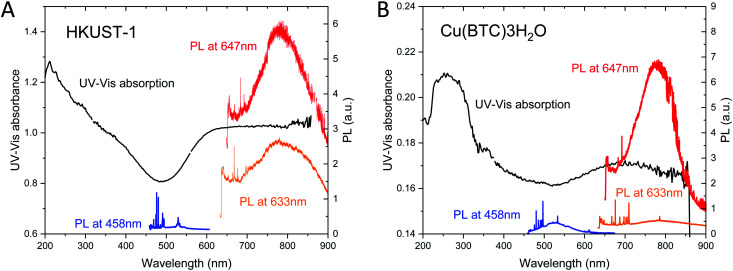
(A) UV-Vis absorption spectrum of HKUST-1 (black curve). (B) UV-Vis absorption spectrum of Cu(BTC)·3H_2_O (black curve). Their photoluminescence (PL) spectra measured at *λ* = 458, 633, and 647 nm are also plotted in blue, orange and red, respectively.

Luminescence is a key property of MOFs that can lead to their potential applications in optoelectronic devices.^50–52^ The luminescence from HKUST-1 and Cu(BTC)·3H_2_O single crystals has been examined using seven different excitation lasers. The photoluminescence spectra of HKUST-1 and Cu(BTC)·3H_2_O crystals measured at laser wavelengths *λ* = 458, 488, 514.5, 531, 568, 633 and 647 nm are shown in Fig. 5. All spectra are normalized by the highest Raman peak. The luminescence peak of HKUST-1 appears in the range 675–1000 nm in the spectra measured at *λ* = 633 and 647 nm (Fig. 5A). Similarly, the spectra of Cu(BTC)·3H_2_O recorded at *λ* = 633 and 647 nm exhibit the luminescence peak in the same range 675–1000 nm (Fig. 5B). In contrast to HKUST-1, the other luminescence peak is observed in the range 458–600 nm at *λ* = 458 nm, that can be associated with the optical band gap of the Cu(BTC)·3H_2_O (see the UV-Vis spectra in Fig. 6). In both cases, the luminescence intensity at *λ* = 647 nm increases linearly with the laser power, and no saturation has been observed up to a half milliwatts as shown in the insets of Fig. 5 (see the corresponding power-dependent spectra in Fig. S12A and B in the ESI†). The highly consistent luminescence spectra of HKUST-1 and Cu(BTC)·3H_2_O at *λ* = 647 nm suggest similar types of defects present in both crystals.

Crystallographic defects play vital roles as far as the visible-range optical properties of wide-bandgap MOFs are concerned. Types of defects in HKUST-1 reported thus far include plane dislocations with free COOH groups,^53,54^ dislocation growth spirals,^55,56^ fractures propagating in the crystal interior,^53^ monovalent copper,^54,57,58^ metal vacancy,^59^ linker vacancy,^60^ defective linkers,^61–63^ and temporary defects as Brønsted sites.^64^ Some defects can be caused post synthesis by exposure to moisture.^42,65^ Methods to avoid defects and reconstruct structural defects were reported.^42,66^

Defects can modify the electronic structure of HKUST-1. The reported primary optical band gap of hydrated HKUST-1 is in the range 3–4 eV (approximately 300–400 nm),^57,58,67–70^ which can be blue-shifted in defect-engineered HKUST-1^57^ or by dehydration.^70^

Fig. 6A shows the UV-Vis spectrum of HKUST-1 crystals. It exhibits the absorption at wavelengths below 450 nm and above 550 nm. The former corresponds to the reported band gap of hydrated HKUST-1, while the latter can be associated with defects. This weak low-frequency visible absorption can be associated with d–d transitions at defective copper pairs leading to the blue colour of defective HKUST-1.^58^ This indicates the presence of defects either in the bulk or on the surface of HKUST-1. The luminescence in the wavelength range 675–1000 nm is excited at laser wavelengths of 633 nm (1.96 eV) and 647 nm (1.92 eV) that is in resonance with the optical band gap of defects.^58,70^

Fig. 6B shows the UV-Vis spectrum of Cu(BTC)·3H_2_O crystals. The photoluminescence spectra of Cu(BTC)·3H_2_O measured at *λ* = 458, 633 and 647 nm are superposed onto the UV-Vis absorption spectrum. To the best of our knowledge, there have been no reports of UV-Vis absorption and luminescence spectroscopy on Cu(BTC)·3H_2_O. The UV-Vis spectrum shows the strong UV absorption at wavelengths below 400 nm and the broad absorption above 600 nm. The long-wavelength tail of the UV absorption coincides with the luminescence excited at *λ* = 458 nm. The broad band above 600 nm leads to the luminescence observed at *λ* = 647 nm. The much weaker luminescence at 633 nm as compared with HKUST-1 can be attributed to the lower-wavelength absorption onset.

## 3 Conclusions

The present work has demonstrated that the reaction between copper and benzene-1,3,5-tricarboxylic (BTC) acid can be controlled by adjusting the pH of the precursor solution to obtain millimeter-sized crystals of two different coordination compounds, namely, metal–organic framework HKUST-1 and coordination polymer Cu(BTC)·3H_2_O. Crystals of HKUST-1 can be synthesized in the pH range 0.7–0.9, whereas Cu(BTC)·3H_2_O in the pH range 2.8–3.2. The post-synthesis transformation from HKUST-1 to Cu(BTC)·3H_2_O is possible, which can be useful as means for environmentally-friendly disposal of the MOF. Both species are found to be luminescent. HKUST-1 exhibits luminescence in the range 675–1000 nm that can be associated with crystallographic defects. Cu(BTC)·3H_2_O in turn is luminescent in the ranges 458–600 nm and 675–1000 nm, that are in-line with the UV-Vis absorption spectrum. The present work demonstrates band-gap engineering by the coordination chemistry and defect-induced luminescence in bulky crystals, that in unison will pave the way for optical applications of coordination polymers and MOFs.

## Conflicts of interest

There are no conflicts to declare.

## Supplementary Material

MA-003-D1MA00866H-s001

MA-003-D1MA00866H-s002
