# Supplementary material for: Room temperature synthesis of a luminescent crystalline Cu–BTC coordination polymer and metal–organic framework
Source: Mater Adv. 2021 Nov 22;3(1):224–31. doi: 10.1039/d1ma00866h (PMC8724791; doi:10.1039/d1ma00866h)
Supplement: MA-003-D1MA00866H-s001 [file MA-003-D1MA00866H-s001.pdf]

Electronic Supplementary Material (ESI) for Materials Advances.
This journal is © The Royal Society of Chemistry 2021

Electronic Supplementary Material

Room temperature synthesis of luminescent crystalline Cu-BTC coordination polymer and metal-organic framework

Shiraz Ahmed Siddiqui,[a] **Alexander Prado-Roller,**[b] **and Hidetsugu Shiozawa**[*a,c]

[a]Faculty of Physics, University of Vienna, Boltzmanngasse 5, 1090 Vienna, Austria

[b] Department of Inorganic Chemistry, University of Vienna, Währinger Straße 42, 1090, Vienna, Austria

[c]J. Heyrovsky Institute of Physical Chemistry, Czech Academy of Sciences, Dolejskova 3, 182 23 Prague 8, Czech Republic

[*]To whom correspondence should be addressed; E-mail: hide.shiozawa@jh-inst.cas.cz & hidetsugu.shiozawa@univie.ac.at

1 Synthesis of HKUST-1

Copper nitrate trihydrate ($CuNO_3{\cdot}3H_2O$) (0.241 grams, 1mmol) and benzene-1,3,5-tricarboxylic acid (BTC) (0.210 grams, 1mmol) was dissolved in a mixture of three solvents (4ml deionised water, 4ml N, N-Dimethylformamide anhyrous DMF, and 4ml ethanol) i.e. in a volummetric ratio of 1:1:1. The pH of the resulting mixyure was 1.8-2.0. Without any pH adjustment, after 16-18 hours HKUST-1 crystals (50-55 micrometers) as shown in Fig. S1 were obtained.

Furthermore, using nitric acid to adjust the pH in the range 0.7-0.9, we get large HKUST-1 crystals in 8-10 days as shown in Fig. S2.

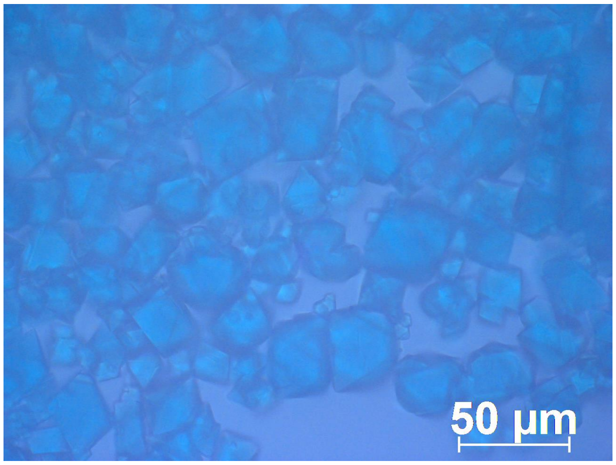

Figure S1: Optical micrograph of the as-synthesized HKUST-1 MOF.

The synthesis of HKUST-1 was also possible using copper (II) chloride ($CuCl_2$) instead of $CuNO_3{\cdot}3H_2O$. The molar ratio of $CuCl_2$ and BTC was kept 1:1 and volummetric ratio of the three solvents(4ml deionised water,4ml DMF and 4ml ethanol) was 1:1:1. The synthesis was successful and we obtained crystals (55-60 micrometers) in 16-18 hours as shown in Fig. S3A.

The next step was to apply our pH-controlled method to obtain large single crystals of HKUST-1. For this, after completely dissolving $CuCl_2$ and BTC (as above) we added 0.1 ml 37% hydrochloric acid (HCl) in the reaction mixture. Large and quality single crystals $\sim$0.9-1.0 mm in size was obtained in 8-10 days as shown in Fig.S3B.

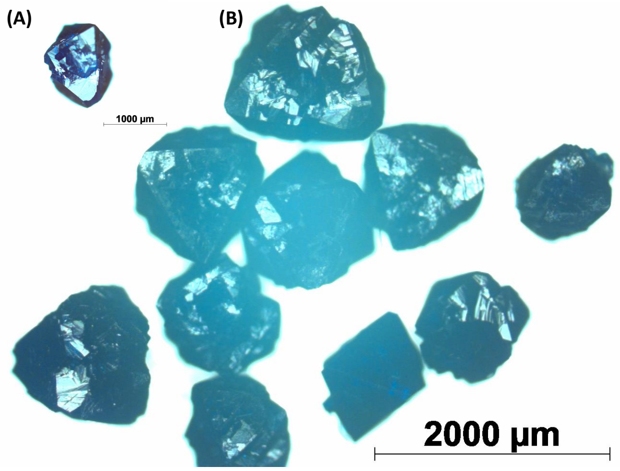

Figure S2: (A) HKUST-1 crystal (pH=0.7-0.9) obtained by pH adjustment using nitric acid reproduced from the main text. (B) large crystals synthesized by pH adjustment using nitric acid.

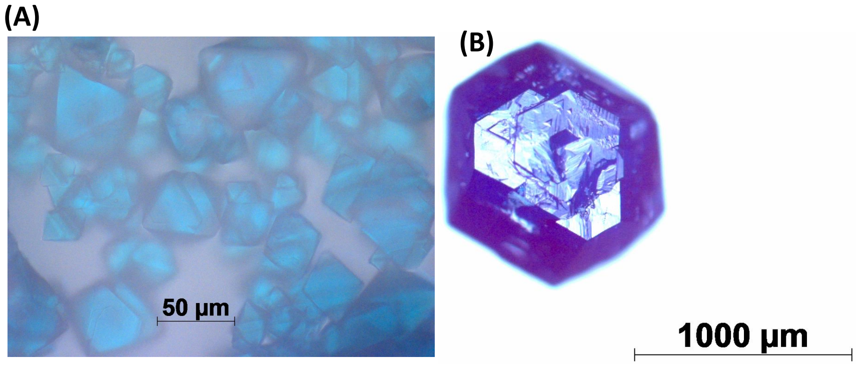

Figure S3: (A) Optical micrograph of the as-synthesized HKUST-1 MOF using $CuCl_2$ without any pH adjustment. (B) optical micrograph of the large single crystal of HKUST-1 synthesized by pH adjustment using HCl.

2 X-ray diffraction analysis

The X-ray intensity data were measured on Bruker D8 Venture diffractometer equipped with multilayer monochromator, Mo K/α INCOATEC micro focus sealed tube and Oxford cooling system. The structures were solved by Charge Flipping and Patterson Methods. Non-hydrogen atoms were refined with anisotropic displacement parameters. Hydrogen atoms were inserted at calculated positions and refined with riding model. The following software was used: Bruker SAINT software package[1] using a narrow-frame algorithm for frame integration, SADABS[2] for absorption correction, OLEX2 for structure solution [3], refinement, molecular diagrams and graphical user-interface, Shelxle for refinement [4] and graphical user-interface SHELXS-2015 for structure solution, SHELXL-2015 for refinement [5], Platon for symmetry check [6]. Experimental data and CCDC-Codes Experimental data (Available online: http://www.ccdc.cam.ac.uk/conts/retrieving.html) can be found in Table S1. Crystal data, data collection parameters, and structure refinement details are given in Tables S2 to S5. Asymmetric Unit, packing views, electron density map and void map visualized in Figs. S4 to S11.

Table S1: Experimental parameter and CCDC-Codes

Sample	Source	Temp.	Detector Distance	Time/ Frame	Frames	Frame width	CCDC
		[K]	[mm]	[s]		[°]	
HKUST-1	Mo	100	40	40	1900	0.360	2069999
Cu(BTC)·3H_2O	Mo	100	40	60	1691	0.360	2070000

2.1 HKUST-1

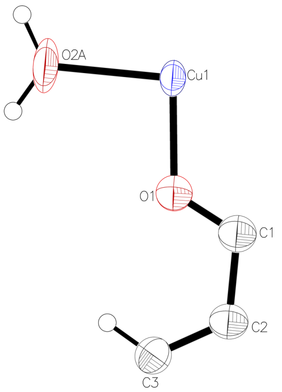

Figure S4: Asymmetric Unit drawn with 50% displacement ellipsoid. The bond precision for C-C single bonds is 0.0083Å. Disorder omitted for clarity.

Table S2: Sample and crystal data

Radiation [Å]	MoKα (λ = 0.71073)	Z	16	Measurement method	\f and \w scans
Crystal habit	clear blue block	a [Å]	26.2775(9)		
Crystal size [mm³]	0.05 × 0.05 × 0.04	b [Å]	26.2775(9)	Abs. correction type	multiscan
Empirical formula	C18H14Cu3O15	c [Å]	26.2775(9)	Abs. correction T_{min}	0.2278
Formula weight [g/mol]	660.91	α [°]	90	Abs. correction T_{max}	0.2550
Temperature [K]	100.0	β [°]	90	Density (calculated) [g/cm³]	0.968
Crystal system	Cubic	γ [°]	90	Absorption coefficient [mm⁻¹]	1.432
Space group	Fm-3m	Volume [Å³]	18144.8(19)	F (000) [e⁻]	5264.0

Table S3: Data collection and structure refinement

2Θ range for data collection [°]	4.384 to 50.638	Index ranges		Goodness-of-fit on F^2	1.109
Reflections collected	92060	h	-31 ≤ h ≤ 31	Diff. peak and hole [e⁻Å⁻³]	0.72/-0.30
Data / restraints / parameters	893/0/39	k	-31 ≤ k ≤ 31		
Refinement method	Charge Flipping	l	-31 ≤ l ≤ 31	Function minimized	$\Sigma w (F_o^2 - F_c^2)^2$
		all data	R1 = 0.0399, wR2 = 0.1163	Weighting scheme	where
		I>2σ(I)	R1 = 0.0363, wR2 = 0.1135	$w=1/[\sigma^2(Fo^2) + (0.0658P)^2 + 60.8619P]$	$P=(F_o^2+2F_c^2)/3$

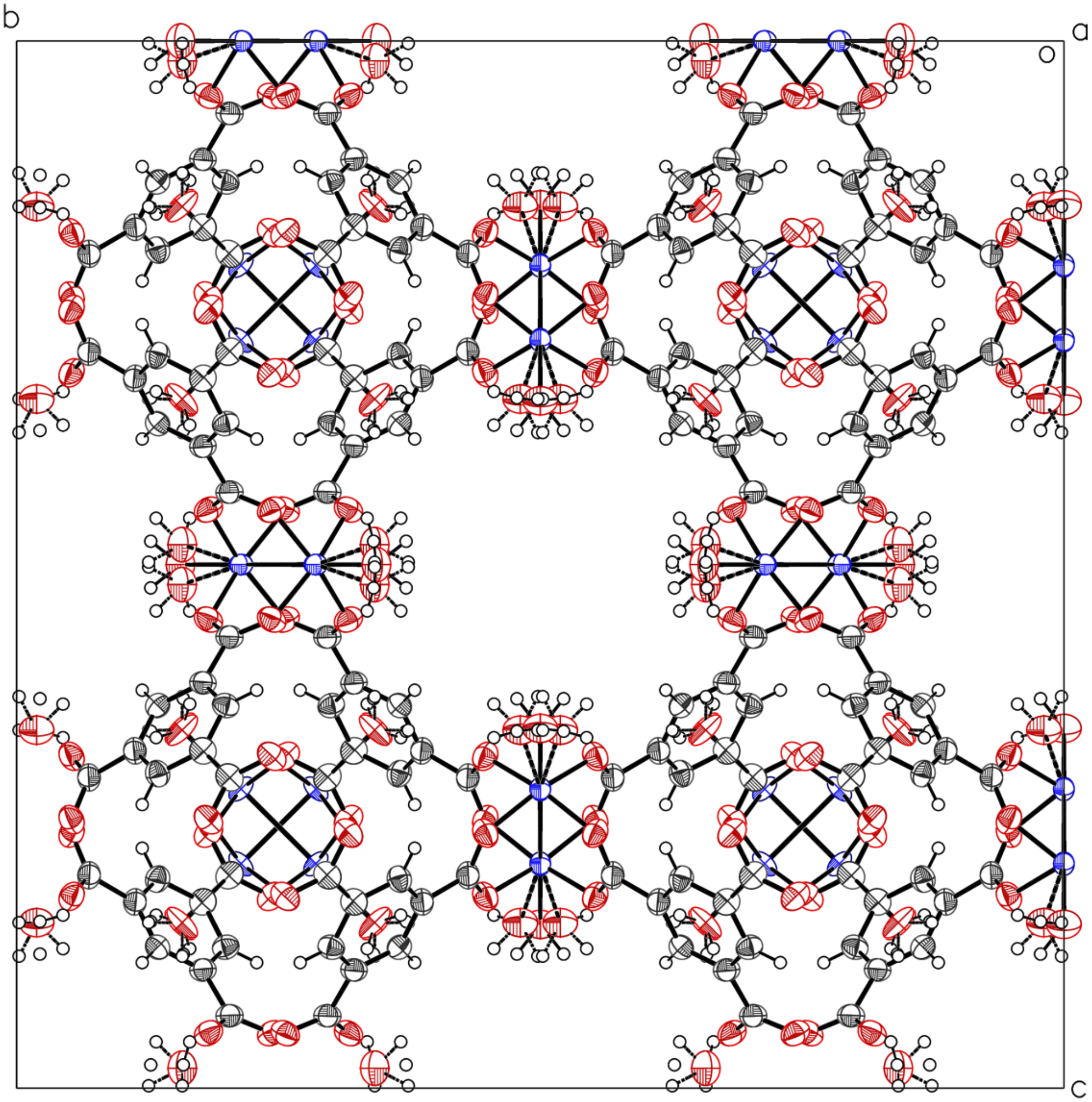

Figure S5: Packing view in the plane b c. Along axis a the void tube in the center is visible enclosed by potential hydrogen bonds forming H_2O positions.

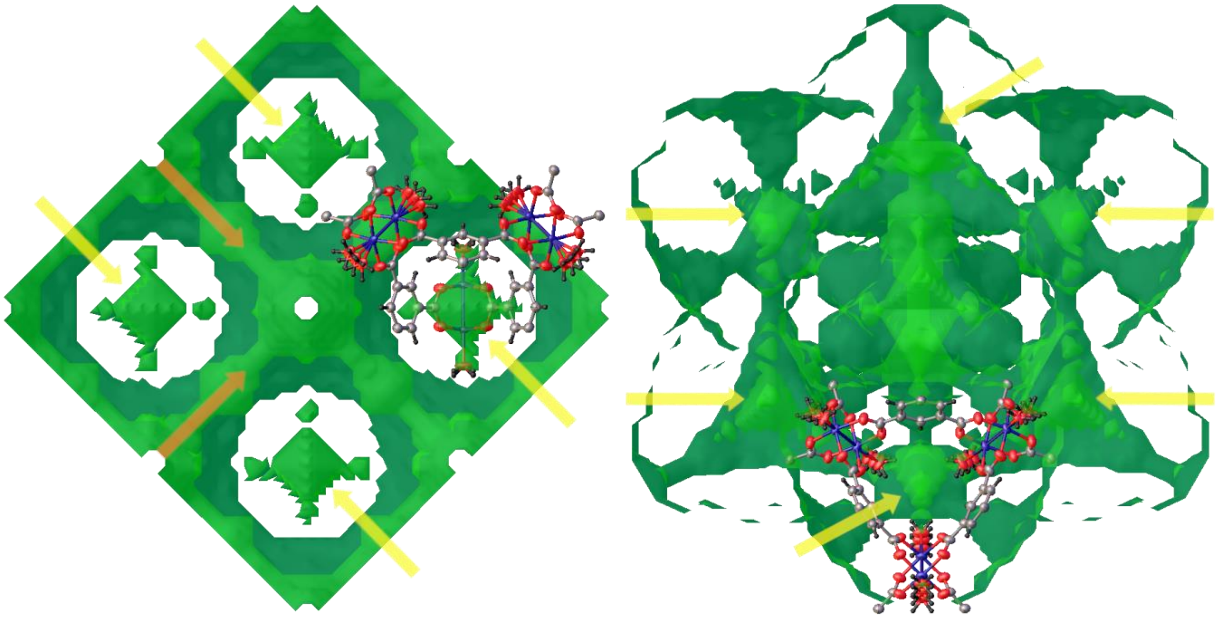

Figure S6: Free solvent accessible voids (left view 100, right view 111). Two different kinds of voids can be characterized. In the left graph we see orange arrows which show the tube voids building one big connected free accessible void for the whole structure. Additionally voids of approximately pyramidal shape are almost separated from the rest of the volume. They are enclosed by planes, simulated by the aromatic rings of the MOF. Pyramid's are pointed out by yellow arrows. Finally we can calculate a volume of 10683 Å^3 and according 2378 e$^-$ per unit cell for the available voids (solvent radius 1.2 Åand truncation 0.7 Å, different to the platon model). This is more than 50% of the unit cell volume of 18145 Å^3. The low height of the separated rest electron densities (smaller than 1.6) forced the decision not to exclude volume with the help of squeeze. The Formula Unit (C18H14Cu3O15) corresponds to 323 e$^-$. The related volume of the unit cell is 668 Å^3 and contains 149 e$^-$ embedded in the free accessible void the values should be similar because of the approximately 50% ration of free void. This causes in fact a lower density, and is detected as an alert by Platon too. But it is a characteristic that should be kept in mind. The effect of the void is bigger in the Platon report because the rest electron density is not part of the calculation in the density

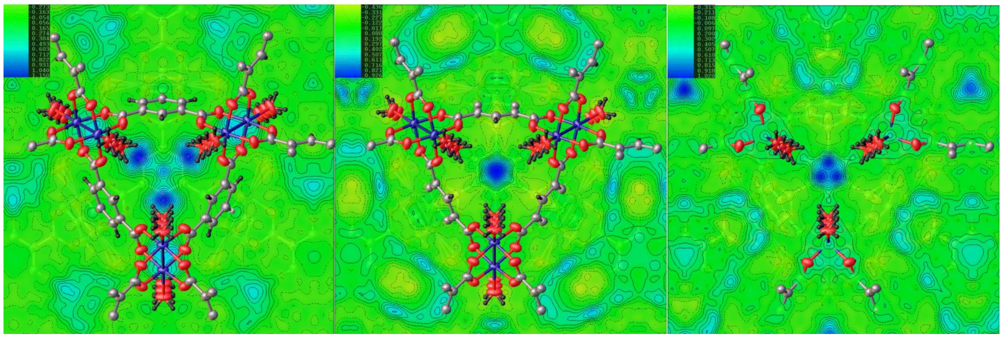

Figure S7: Electron density distribution along view 111 in the pyramidal void. The bottom is formed by the aromatic ring (not visible) the top is formed by the three orientations of disordered water molecules pointing to the center located on Cu. Three main layers of electron densities are visualized. From left to right the layer moves from bottom to top. From the distribution we must conclude that solvent is enclosed in the pyramidal void. The density is not enough to model solvents. It seems that several different positions are possible

2.2 Cu(BTC)·3H_2O

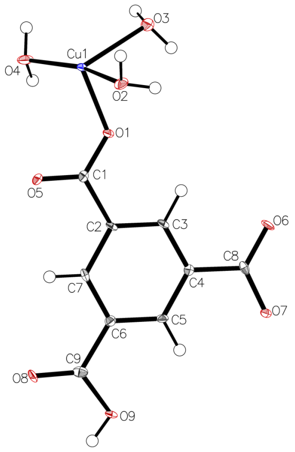

Figure S8: Asymmetric Unit drawn with 50% displacement ellipsoid. The bond precision for C-C single bonds is 0.0050Å.

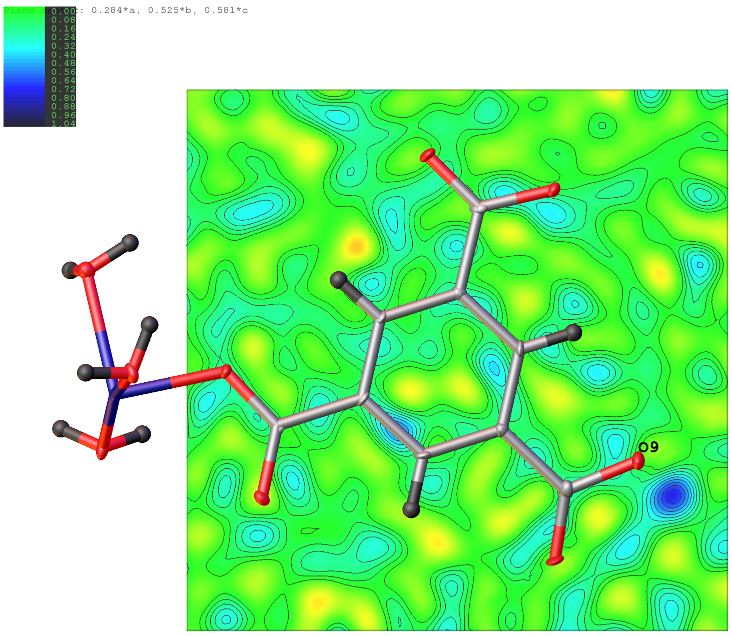

Figure S9: Density map showing that one of the carboxyl groups is protonated.

Table S4: Sample and crystal data

Radiation [Å]	MoKα (λ = 0.71073)	Z	4	Measurement method	\f and \w scans
Crystal habit	clear blue block	a [Å]	6.7654(11)		
Crystal size [mm^3]	0.035 × 0.035 × 0.035	b [Å]	18.813(5)	Abs. correction type	multiscan
Empirical formula	C9H10CuO9	c [Å]	8.5144(13)	Abs. correction Tmin	0.2237
Formula weight [g/mol]	325.71	α [°]	90	Abs. correction Tmax	0.2657
Temperature [K]	100.0	β [°]	92.439(5)	Density (calculated) [g/cm3]	1.998
Crystal system	Monoclinic	γ [°]	90	Absorption coefficient [mm^{-1}]	2.062
Space group	P21/n	Volume [Å^3]	1082.7(4)	F (000) [e$^-$]	660.0

Table S5: Data collection and structure refinement

2Θ range for data collection [°]	4.33 to 50.698	Index ranges		Goodness-of-fit on F^2	1.050
Reflections collected	19273	h	-8 ≤ h ≤ 8	Diff. peak and hole [e$^-$Å^{-3}]	0.46/-0.58
Data / restraints / parameters	1982/0/170	k	-22 ≤ k ≤ 22		
Refinement method	Patterson Method	l	-10 ≤ l ≤ 10	Function minimized	Σ w (F_o^2 - F_c^2)^2
		all data	R1 = 0.0509, wR2 = 0.0838	Weighting scheme	where
		I>2σ(I)	R1 = 0.0338, wR2 = 0.0782	w=1/[σ^2(Fo^2) + 1.8669P]	P=(F_o^2+2F_c^2)/3

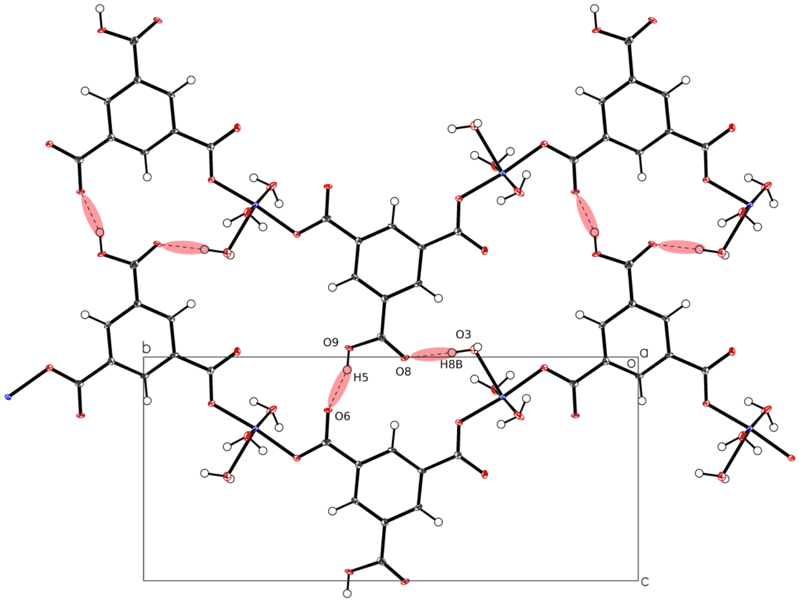

Figure S10: Packing in the plane bc. Moderate hydrogen bonds form a two dimensional net in bc

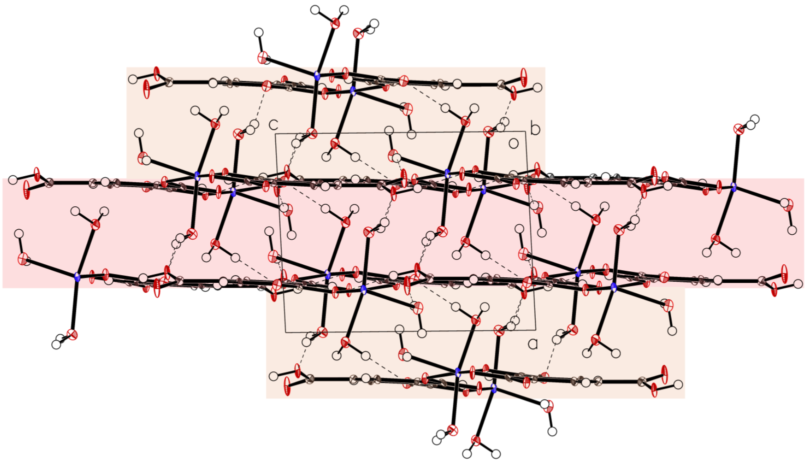

Figure S11: Packing in the plane ac. The different layers are connected via moderate hydrogen bonds again. Additionally to the chain structure of the molecule a three dimensional network of hydrogen bonds characterizes the structure.

3 Photoluminescence

The luminescence spectra of HKUST-1 and Cu(BTC)·3H_2O measured with a laser wavelength of 647 nm and different laser powers are shown in Fig. S12 A and B.

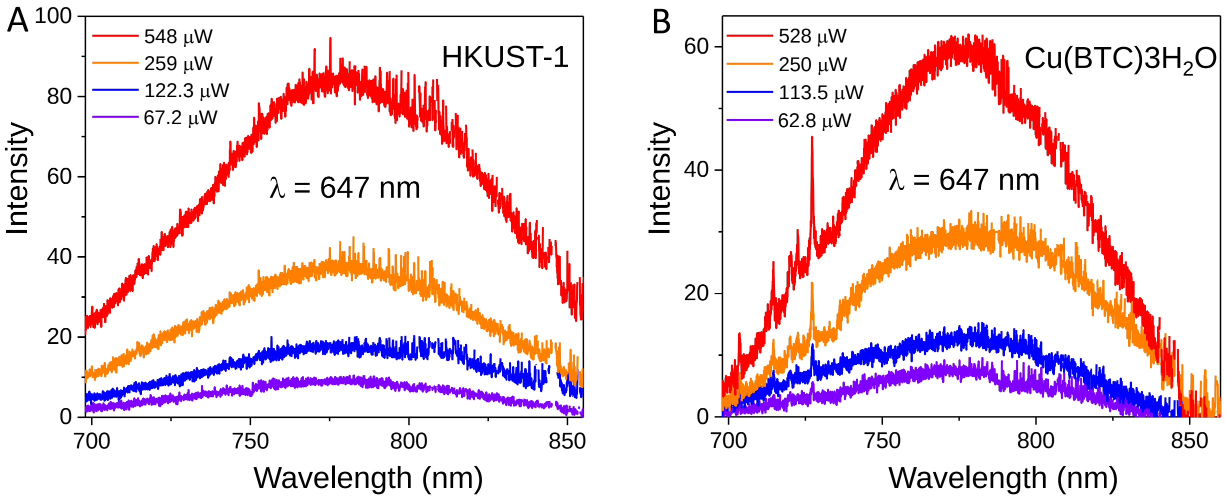

Figure S12: (A) Photoluminescence spectra of HKUST-1 measured at a laser wavelength of 647 nm and powers of 67.2, 122.3, 259 and 548 μW. (B) Photoluminescence spectra of Cu(BTC)·3H_2O measured at a laser wavelength of 647 nm and powers of 62.8, 113.5, 250 and 528 μW.

The luminescence spectra of Cu(BTC)·3H_2O measured with a 458 nm laser at different laser powers are shown in Fig. S13. The relationship between the photoluminescence intensity and excitation power is shown in the inset, which demonstrates that the intensity is linear as a function of laser power and no saturation is observed up to 356 μW.

After being exposed to a 633 nm laser at high powers, the crystal surface of HKUST-1 appears to get damaged, as observed as the dark spot within the red circle in the inset micrograph in Fig. S14 and as the spectral changes at laser powers of 81.2 and 172 μW in Fig. S14. No laser damages were observed with 458 and 633 nm lasers.

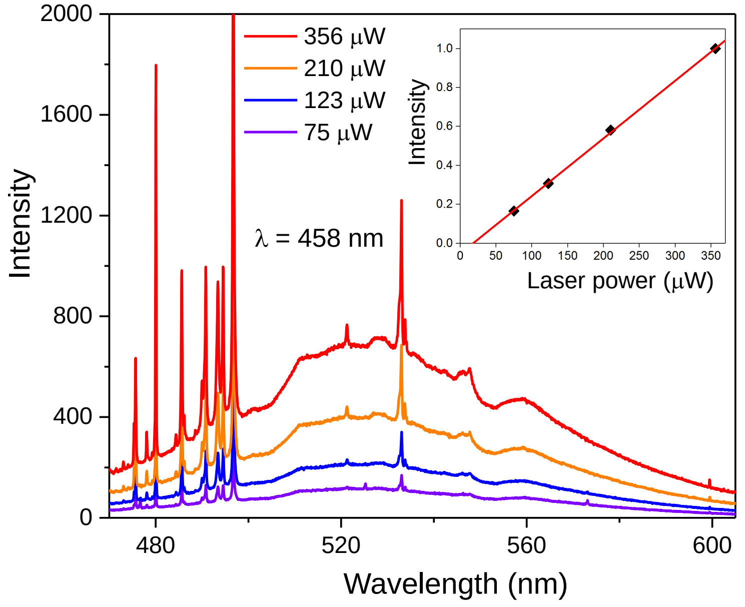

Figure S13: Photoluminescence spectra of Cu(BTC)·3H_2O measured using a 458 nm laser at laser powers of 75, 123, 210 and 356 μW. The inset shows the photoluminescence intensity versus the laser power.

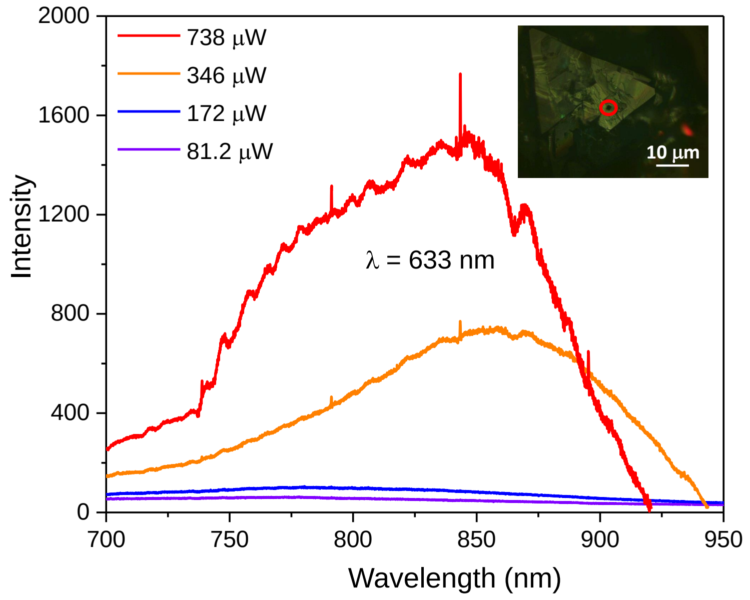

Figure S14: Photoluminescence spectra of HKUST-1 measured using a 633 nm laser at laser powers of 81.2, 172, 346 and 738 μW. The inset is the micrograph of the crystal after the measurements. The laser damage is observed as the dark spot within the red circle.

4 Infrared absorption

Figure S15 shows the O-H stretching mode of the coordinated water observed for HKUST-1 and Cu(BTC)·3H_2O. Both peaks are located at $\sim$ 3675 cm^{-1} and skewed towards lower wavenumbers. The spectra are consistent with those previously reported for HKUST-1 [7, 8, 9, 10, 11, 12, 13, 14, 15, 16, 17] and for Cu(BTC)·3H2O [18]. The broader peak for Cu(BTC)·3H_2O than that for HKUST-1 can be attributed to different coordination environments. In HKUST-1, two water molecules are coordinated symmetrically to the dimeric copper core in the paddlewheel structure. In Cu(BTC)·3H_2O, three water molecules are coordinated to copper. The coordination axis of one is in a direction along the 2D layer of BTC, while that of the other two are out of the layer.

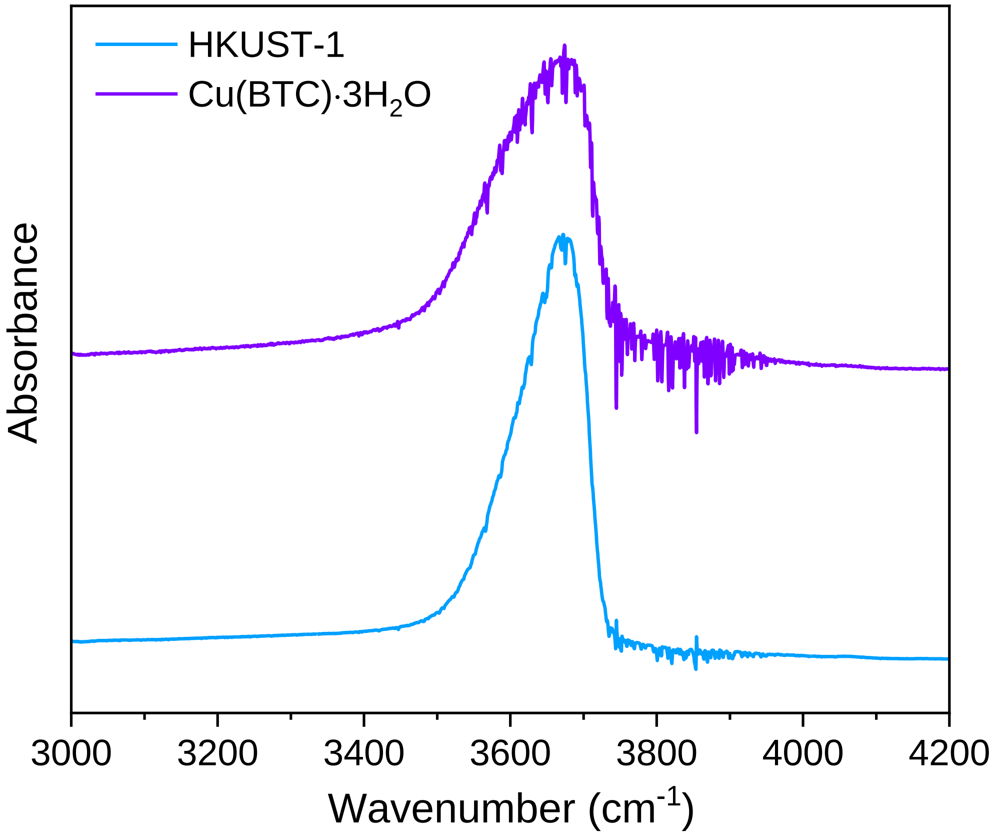

Figure S15: Infrared spectra of HKUST-1 (bottom) and Cu(BTC)·3H_2O (top).

References

[1] Bruker saint v8.38b copyright © 2005-2019 bruker axs.

[2] George M Sheldrick. Program for empirical absorption correction of area detector data. *SADABS*, 1996.

[3] Oleg V. Dolomanov, Luc J. Bourhis, Richard J. Gildea, Judith A. K. Howard, and Horst Puschmann. OLEX2: a complete structure solution, refinement and analysis program. *JOURNAL OF APPLIED CRYSTALLOGRAPHY*, 42:339–341, APR 2009.

[4] Christian B. Huebschle, George M. Sheldrick, and Birger Dittrich. ShelXle: a Qt graphical user interface for SHELXL. *JOURNAL OF APPLIED CRYSTALLOGRAPHY*, 44(6):1281–1284, DEC 2011.

[5] George M. Sheldrick. SHELXT - Integrated space-group and crystal-structure determination. *ACTA CRYSTALLOGRAPHICA A-FOUNDATION AND ADVANCES*, 71(1):3–8, JAN 2015.

[6] Anthony L. Spek. Structure validation in chemical crystallography. *ACTA CRYSTALLOGRAPHICA SECTION D-STRUCTURAL BIOLOGY*, 65(2):148–155, FEB 2009.

[7] Gerardo Majano, Oliver Martin, Markus Hammes, Stef Smeets, Christian Baerlocher, and Javier Perez-Ramirez. Solvent-Mediated Reconstruction of the Metal-Organic Framework HKUST-1 (Cu-3(BTC)(2)). *ADVANCED FUNCTIONAL MATERIALS*, 24(25):3855–3865, JUL 2 2014.

[8] Kun-Yi Andrew Lin and Yu-Tsung Hsieh. Copper-based metal organic framework (mof), hkust-1, as an efficient adsorbent to remove p-nitrophenol from water. *Journal of the Taiwan Institute of Chemical Engineers*, 50:223–228, 2015.

[9] Rebeca Yepez, Salvador García, Persi Schachat, Manuel Sánchez-Sánchez, Juan H González-Estefan, Eduardo González-Zamora, Ilich A Ibarra, and Julia Aguilar-Pliego. Catalytic activity of hkust-1 in the oxidation of trans-ferulic acid to vanillin. *New Journal of Chemistry*, 39(7):5112–5115, 2015.

[10] W. W. Lestari, R. E. Nugraha, I. D. Winarni, M. Adreane, and F. Rahmawati. Optimization on electrochemical synthesis of hkust-1 as candidate catalytic material for green diesel production. *AIP Conference Proceedings*, 1725(1):020038, 2016.

[11] Feng Xu, Ying Yu, Jian Yan, Qibin Xia, Haihui Wang, Jing Li, and Zhong Li. Ultrafast room temperature synthesis of gro@hkust-1 composites with high co2 adsorption capacity and co2/n2 adsorption selectivity. *Chemical Engineering Journal*, 303:231–237, 2016.

[12] Feroz Ahmad Sofi, Kowsar Majid, and Owais Mehraj. The visible light driven copper based metal-organic-framework heterojunction:hkust-1@ag-ag3po4 for plasmon enhanced visible light photocatalysis. *Journal of Alloys and Compounds*, 737:798–808, 2018.

[13] Witri Wahyu Lestari, Irwinsyah, Teguh Endah Saraswati, Yuni Krisyuningsih Krisnandi, Ubed Sonai Fahrudin Arrozi, Eddy Heraldy, and Grandprix T. M. Kadja. Composite Material Consisting of HKUST-1 and Indonesian Activated Natural Zeolite and its Application in CO2 Capture. *OPEN CHEMISTRY*, 17(1):1279–1287, JAN 2019.

[14] Aili Yang, Ping Li, and Jingrong Zhong. Facile preparation of low-cost hkust-1 with lattice vacancies and high-efficiency adsorption for uranium. *RSC Adv.*, 9:10320–10325, 2019.

[15] Mohsen Zarei Mohammadabad, Mazaher Moeinaddini, Mohsen Nowrouzi, Reza Rafiee, and Alireza Abbasi. Facile and cost-efficient synthesis of highly efficient co 2 adsorbents: a pathway towards a green environment. *Journal of Porous Materials*, 27(6):1659–1668, 2020.

[16] Francesco Silvio Gentile, Marianna Pannico, Mauro Causa, Giuseppe Mensitieri, Giulio Di Palma, Giuseppe Scherillo, and Pellegrino Musto. Metal defects in HKUST-1 MOF revealed by vibrational spectroscopy: a combined quantum mechanical and experimental study. *JOURNAL OF MATERIALS CHEMISTRY A*, 8(21):10796–10812, JUN 7 2020.

[17] Charles O Oseghale, Elias E Elemike, Ayi A Ayi, Ayomide H Labulo, Elijah T Adesuji, and Benjamin Orimolade. Thermolytic conversion of copper (ii) based coordination polymer into copper oxide–carbon nanocomposite for selective removal of cd (ii) from aqueous solution. *Journal of Cluster Science*, 32(2):319–326, 2021.

[18] Abdul Rauf, Junwei Ye, Siqi Zhang, Ye Qi, Guangyao Wang, Ying Che, and Guiling Ning. Copper(ii)-based coordination polymer nanofibers as a highly effective antibacterial material with a synergistic mechanism. *Dalton Trans.*, 48:17810–17817, 2019.
